# Mutation Breeding in Tomato: Advances, Applicability and Challenges

**DOI:** 10.3390/plants8050128

**Published:** 2019-05-14

**Authors:** Juhi Chaudhary, Alisha Alisha, Vacha Bhatt, Sonali Chandanshive, Nirbhay Kumar, Zahoor Mir, Ashwini Kumar, Satish K. Yadav, S. M. Shivaraj, Humira Sonah, Rupesh Deshmukh

**Affiliations:** 1 Department of Biology, Oberlin College, Oberlin, OH 44074, USA; juhi.chaudhary@gmail.com; 2 National Agri-Food Biotechnology Institute (NABI), Mohali, Punjab 140308, India; alishaalisha001@gmail.com (A.A.); vacha.biotech@gmail.com (V.B.); chandanshivesonali444@gmail.com (S.C.); nirbhay.kumar@imsuc.ac.in (N.K.); 3 National Research Center on Plant Biotechnology, New Delhi, Delhi 110012, India; zahoorbio@gmail.com; 4 Division of Plant Pathology, ICAR-IARI, New Delhi, Delhi 110001, Inida; ashwinikumar1500@gmail.com; 5 National Bureau of Plant Genetic Resources, New Delhi, Delhi 110012, India; satish.yadav1@icar.gov.in; 6 Faculté des sciences de l’agriculture et de l’alimentation (FSAA), Université Laval, Quebec, QC G1V 0A6, Canada; sraj100@gmail.com

**Keywords:** genome-editing, mutagenesis approaches, mutmap, mutation breeding, next generation sequencing tools, tomato

## Abstract

Induced mutagenesis is one of the most effective strategies for trait improvement without altering the well-optimized genetic background of the cultivars. In this review, several currently accessible methods such as physical, chemical and insertional mutagenesis have been discussed concerning their efficient exploration for the tomato crop improvement. Similarly, challenges for the adaptation of genome-editing, a newly developed technique providing an opportunity to induce precise mutation, have been addressed. Several efforts of genome-editing have been demonstrated in tomato and other crops, exploring its effectiveness and convenience for crop improvement. Descriptive data compiled here from such efforts will be helpful for the efficient exploration of technological advances. However, uncertainty about the regulation of genome-edited crops is still a significant concern, particularly when timely trait improvement in tomato cultivars is needed. In this regard, random approaches of induced mutagenesis are still promising if efficiently explored in breeding applications. Precise identification of casual mutation is a prerequisite for the molecular understanding of the trait development as well as its utilization for the breeding program. Recent advances in sequencing techniques provide an opportunity for the precise detection of mutagenesis-induced sequence variations at a large scale in the genome. Here, we reviewed several novel next-generation sequencing based mutation mapping approaches including Mutmap, MutChromeSeq, and whole-genome sequencing-based mapping which has enormous potential to accelerate the mutation breeding in tomato. The proper utilization of the existing well-characterized tomato mutant resources combined with novel mapping approaches would inevitably lead to rapid enhancement of tomato quality and yield. This article provides an overview of the principles and applications of mutagenesis approaches in tomato and discusses the current progress and challenges involved in tomato mutagenesis research.

## 1. Introduction

Tomato (*Solanum lycopersicum* L.) is one of the most popular cultivated vegetable crops worldwide. It is a model plant of the Solanaceae family because of its short life cycle, simple diploid genome, availability of efficient tools for plant transformation, and available genome sequence [1,2]. The global demand for tomato increased tremendously in recent years due to its diverse utility in raw, cooked, and processed food as well as its nutritional value. In addition, the changes in climatic conditions and human population growth etc. are posing the biggest challenge to sustain the supply worldwide. This necessitates the sustainable production of nutritious and high-yielding tomato cultivars considering the rapidly changing environmental conditions. The development of high yielding cultivars with improved fruit quality and tolerance against abiotic and biotic stresses is challenging, mostly due to the narrow genetic diversity existing in the cultivated tomatoes. To overcome the bottleneck, efforts are being made to explore wild species like *Solanum pimpinellifolium* (which has only 0.6% nucleotide divergence from cultivated tomato) [3]. However, introgression of the wild genome considerably hampers the well optimized high-yielding genetic background of the commercial tomato cultivars. In addition, introgression breeding is a time-consuming process. Besides, crossing incompatibility of cultivated varieties with wild species is also a limitation. In this regard, mutation breeding provides one of the most promising option to broaden the genetic diversity and achieve rapid crop improvement. Induced mutagenesis has been performed in a number of crop species including rice [4], banana [5], and watermelon [6]. 

The change in climatic conditions are unpredictable, therefore there is a need for new varieties to be developed regularly for sustainable production. Since the spontaneous mutation rate is very slow, induced mutation is necessary to enhance the rate of genetic diversity so that breeders can exploit the diverse varieties in plant breeding programs. In addition, multiple-trait mutants can be isolated by mutation breeding and the chances of survival of mutant varieties are much higher under rapidly fluctuating climatic conditions. Mutagenesis is an efficient process of generating mutation, which can occur spontaneously or can be induced by a mutagen. Several efficient methods to induce genetic mutations have been developed which are broadly classified as physical and chemical mutagenesis based on the nature of mutagenic agent. Mutagens provide better chances to obtain desirable phenotypic variation and they are also used to study genotypic variations associated with phenotypes as well as annotation of gene function. A number of studies on mutagenesis in tomato have been performed to discover the function of genes associated with economically important traits like fruit quality (Table 1). Several physical and chemical mutagenic agents like gamma rays and ethyl methane sulfonate (EMS) have been used in tomato for induced mutagenesis (Table 1). Numerous genetic resources of tomato mutant lines have been generated worldwide by using EMS, gamma-rays and fast neutron mutagenesis. Additionally, several resources to find a variety of tomato mutants such as LycoTILL for Red Setter, ‘Genes that make tomatoes’ for M82, and TOMATOMA for Micro-Tom, are publicly available. The conventional physical and chemical mutagenesis approaches induce random mutations in the genome. Hence, it leads to several non-target mutations, so it is difficult to obtain the desired one. However, newly developed mutagenesis approaches based on the genetic engineering tools are very specific to alter the target gene. Currently, the most commonly used approach of targeted mutagenesis is gene editing by CRISPR/Cas9 and TALENs due to the availability of genomic sequences of *S. lycopersicum* and its wild relative *S. pimpinellifolium* [7,8]. 

Generating a large mutant population and subsequent screening for phenotypic changes is comparatively easier than the identification of casual variation induced by the mutagenesis. Conventionally, linkage analysis using molecular markers and segregating the population developed from the mutant line and genotype with diverse genetic background have been used for the mapping of the casual variation. Even after genetic mapping, extensive efforts are required to pinpoint the casual variation. Fortunately, the entire genome sequence is available for tomato, which provides the basis for the mapping and identification of variations. The whole genome sequencing data have led to the identification of millions of single nucleotide polymorphisms (SNPs) and indels (insertions and deletions) in tomato genotypes and mutant lines. Considering the development in the high-throughput next generation sequencing (NGS) technology, several novel approaches have been developed for the identification of mutations in the mutagenized populations. MutMap (mapping-by-sequencing) and MutChromSeq approach are the notable examples where NGS technology is used to identify the casual variation induced by the mutagenesis. MutChromSeq helps to identify the candidate genes in shortest time and has been employed successfully in barley and wheat. However, it has not been utilized in tomato for the identification of candidate genes. In this review, we discuss the various induced mutagenesis approaches, their utility for tomato improvement, available mutant resources and mutation mapping methods to provide an insight into the advances in plant mutagenesis research.

## 2. Induced Mutagenesis

### 2.1. Chemical Mutagenesis

Chemical mutagenesis is one of the most efficient and convenient approaches used in diverse plant species. In tomato, EMS and sodium azide have been used as chemical mutagens (Table 1). The EMS is the most widely used chemical mutagen in plants because of its high effectiveness at inducing point mutations and deletions in the chromosomal segments [12]. In tomato, EMS has been shown to produce both morphological variations and desired trait improvements like disease resistance, fruit quality, and male sterility [16,17]. In several cases, EMS mutagenesis found efficient where other conventional tomato breeding approaches are not feasible, for instance, in the case of Broomrape (*Orobanche ramosa* L.) a wide parasitic weed responsible for the large economic losses in tomato yield [12]. No resistance source is known therefore to overcome the limitation, EMS mutagenesis has been used and after the multiple screening of offspring from several generations six resistance lines against the Broomrape have been developed [12]. Similarly, EMS mutagenesis has been used to develop resistance against potyvirus, a widespread destructive virus for tomato. It has been shown that mutation in translation initiation factors mainly eukaryotic initiation factor 4E (eIF4E) plays a role in tomato resistance to the two potyviruses namely *Potato virus Y* (PVY) and *Tobacco etch virus* (TEV). The recessive resistance gene *pot-I* encodes eIF4E 1 protein and the difference between resistance and susceptible proteins was found to be the substitution of four amino acids. So, mutagenesis of the M82 variety was performed with EMS and a mutant population was developed; subsequent screening identified the mutant line with a spliced variant of eIF4E 1 providing resistance to both the potyvirus strains in tomato [15].

Conventional mutation techniques helped in both forward as well as reverse genetic studies [18]. The reverse genetics tool, targeted induced local lesions in genomes (TILLING) has been developed to identify allelic variation of mutants efficiently [18]. The TILLING approach have been used to screen mutant population developed EMS and fast neutron mutant to enhance the genomics studies in tomato [14]. Efforts have been made to raise mutant populations from tomato cultivars Red Setter, TPAADASU, and Micro-tom using the EMS mutagen. Among different cultivars, a mutant population derived from Micro-tom has great importance since it is considered as a model plant due to its small size, fast growth and ease of transformation, and is being used in large-scale mutant screening [19]. Furthermore, TILLING platforms have been developed from these mutant populations which will surely facilitate the reverse genetics studies in tomato [1].

### 2.2. Physical Mutagenesis

Physical mutagens such as fast neutron and gamma rays produce vast amount genetic variability and have played a significant role in plant genetics studies. In tomato, physical mutagens including radiations like gamma rays and ionization with neutrons are frequently employed for mutagenesis (Table 1). For example, about 6000 Micro-Tom mutant lines were generated using gamma-rays irradiation. From this, around 24 morphological mutant lines and eight brix mutant lines were screened and QTL analysis revealed the involvement of two loci in the brix mutants which can be used to identify brix-regulating genes in tomato [13]. Similarly, fast neutron and other physical mutagens can be explored to induce beneficial variability in tomato for enhanced quality and yield. Besides, physical mutagens are not as popular as chemical mutagens mostly due to the requirement of specialized instruments, skilled staff and highly secure laboratories. Physical mutagens like gamma-irradiation produces severe genetic mutations due to large chromosomal deletions and reconstitution of chromosome [13].

Fast neutron mutagenesis is relatively new for plant science and the least explored approach [20]. However recent studies have shown the effectiveness and ease of reverse genetics using a fast neutron mutagenesis population. Fast neutron bombardment is used to generate deletions and chromosomal rearrangements in the genome randomly and hence employed for the identification and isolation of mutants in target genome [20,21]. The major fast neutron technique used in plants is Deletagene. In this method, fast neutrons are used to induce mutations in the seeds and the deletions produced are further analysed by polymerase chain reaction (PCR) using specific primers flanking the target genes [20,21].

### 2.3. Insertional Mutagenesis

Gene inactivation has been successfully employed in determining the function of unknown genes in several plant species. Inactivation of endogenous genes has been employed by introducing sense and antisense copies of a target gene; however, when the target locus is uncharacterized, insertional mutagenesis is considered the most convenient approach of gene inactivation [22,23]. The insertional mutagenesis is performed by using a DNA sequence (T-DNA, transposon or retrotransposon) to mutate and tag the gene, which can be studied by observing the mutated site using the tag as an identifier. Furthermore, a PCR-based approach, site-selected insertion (SSI), is used for detection of mutations in known genes by insertional inactivation. In this technique, two primers are designed, one specific for sequences in the target gene and another for sequences in the transposon, and amplification products are observed for genes with an insertion. This technique was successfully used to detect the transpositions that occurred in *polygalacturonase* (PG) and *dihydroflavonol 4-reductase* (DHFR) genes by *Ds1* elements. It was shown that SSI and maize Ds elements significantly facilitate insertional mutagenesis in tomato [24]. In another study conducted by the same group, SSI was used to generate insertional mutations in *PG* gene to delay fruit ripening in tomato. Among 4000 progenies screened for insertions in *PG*, five were found to be germinally transmitted *Ds* insertions and one among them revealed to have two *Ds* insertions. Around 1000-fold polygalacturonase reduction have been observed in these mutants. The Ds elements were further stabilized by eliminating transposase so that no genetic material is left in the PG gene and this could be exploited on a commercial scale to generate desired mutations in plants with no transgenic material [25]. Moreover, a significant number of studies have been performed for morphological and functional mutation induction by both transposons and T-DNA in tomato (Table 2).

### 2.4. Mutagenesis by Antisense Approach

Antisense technology is performed to ‘knock down’ the genes in order to study their function. It is performed in three different ways; one is by binding the single stranded antisense complementary nucleic acid sequence to target sense mRNA to block its translation while another is by binding catalytically active oligonucleotides like ribozymes which degrade specific RNA sequences. The third method is by RNA interference in which small interfering RNA (siRNA) helps in the cleavage of RNA by formation of RNA induced silencing complex (RISC). In tomato, several studies have been performed using antisense approach, for example, the *entire* (*e*) locus which is known to control leaf morphology and responsible for the conversion of compound leaf morphology to simple leaves. Research has indicated that SlIAA9 is involved in tomato fruit development and leaf morphogenesis. The antisense mutagenesis approach was utilized to generate transgenic plants against SlIAA9 gene to understand the mechanism of compound leaf conversion. The leaf morphology of transgenics and *entire* locus mutants were found to be similar and no functional redundancy was found for the SlIAA9 gene in tomato. Additionally, a single-base cytosine deletion was found in coding region of SlIAA9 of *entire* mutants and mapping analysis revealed that both SlIAA9 and e genes were located on chromosome 4 [30].

Tomato breeders use *rin* (ripening inhibitor) mutation to reduce the softening of fruits. This mutation produced firm fruits with delayed ripening effects but decreased nutritional value, color and flavor of the tomato. Nonetheless, the tomato genome sequencing helped to find a number of genes regulating the ripening process and PL was found to be one of the enzymes involved in the change of texture in the tomato fruits. Therefore, in an attempt to delay the ripening of fruits, cv. Ailsa Craig was chosen. Furthermore, real time expression analysis revealed the expression of five PL genes, but one was found to be highly expressed and was selected as a target. So, RNAi construct was made against this gene and the transgenic tomato fruits obtained showed improved shelf life of storage for 14 days at 20 °C. Furthermore, silencing this gene improved the fruit firmness without affecting color, yield, weight, taste, aroma or total soluble solids [31]. In another study, silencing the PL gene by antisense inhibition in tomato has been reported to play an important role in fruit softening and pathogen resistance. A total of 22 PLs were identified in tomato and silencing of dominantly expressive gene SIPL (Solyc03g111690) resulted in enhanced fruit firmness as well as pathogen-resisting ability [32]. In conclusion, the use of the antisense approach has significantly enhanced the understanding of tomato fruit development and ripening mechanism.

### 2.5. Mutagenesis by Genome-Editing Approaches

The ability to precisely edit genomes is a promising approach for advancing both basic and applied plant research. Recently, the development of sequence-specific DNA nucleases has been widely used for targeting specific genes to improve the productivity of important crop plants. Tomato is reported to be an ideal crop for genome editing in plants due to the availability of its genome sequence, ease of transformation and its diploidy nature [7,33,34,35].

The three principle genome-editing techniques that are used to target genes in DNA specific manner include zinc finger nucleases (ZFNs), transcription activator like effector nucleases (TALENS) and clustered regularly interspaced short palindromic repeats (CRISPR)/CRISPR-associated9 (Cas9) endonuclease. 

### 2.6. Genome Editing by Zinc Finger Nucleases (ZFNs)

Zinc finger nucleases (ZFNs)-based genome editing is one of the primitive technologies which makes it possible to perform precise site-specific mutations [36]. The ZFNs form dimmers which recognize a specific target site and make a double-strand DNA break [36]. Subsequently, the endogenous DNA repair system act on the resultant double-strand DNA break to repair it by non-homologous end joining (NHEJ) or a homology-directed (HR) repair mechanism. At the target site, the repair mechanism leads to variations like insertions, deletions and single nucleotide polymorphisms (SNPs). The ZFN approach have been used in several plant species including tomato [37]. In a seminal study by Hilioti et al. (2016), the effectiveness of the ZFN approach was shown to create a variation in a *LEAFY-COTYLEDON1-LIKE4* (*L1L4*) gene [37]. Subsequent development in other convenient genome-editing approaches as described below, the use of ZFN has become less frequent. 

### 2.7. Genome Editing by Transcription Activator-Like Effector Nucleases (TALENs)

Transcription activator-like effector nucleases (TALENs) are composed of a free designable DNA, and have been successfully employed for specific gene mutation in many plant species including *Arabidopsis, Brachypodium*, barley, rice, tobacco, wheat, and soybean. The first successful report on gene editing in tomato by TALENs was reported in 2014 [8]. DELLA proteins are reported to be negative regulator of GA signaling and PROCERA (PRO) is the only reported DELLA gene in tomato. Previous reports to determine the role of PRO in GA signaling by missense mutation in the gene resulted in partial loss of activity. Therefore, targeted mutagenesis of PRO by TALENs was performed by Lor, Starker, Voytas, Weiss and Olszewski [8]. About 15% of the plants carried *pro* alleles and these mutants showed increased levels of GA. In addition, the mutant plants showed altered phenotypes like long internode length, smooth leaf margins and light green vegetation. A truncated PRO protein was produced due to frame-shift mutations and these mutations were also showed to be heritable [8]. Another report by Čermák et al. (2015) compared genome editing efficiency of TALENS and CRISPR/Cas9 approaches in tomato by targeting Anthocyanin mutant 1 (ANT1) gene. In this study, geminivirus replicons were used to create mutations and two-thirds of insertions were found to be precise without any unexpected sequence modifications in the tomato genome [38]. Hence, studies demonstrated TALENs as an efficient tool for creating targeted mutations in tomato. 

### 2.8. Gene Editing by CRISPR/Cas9

Until 2013, ZFNs and TALENs were used for the precise genome-editing. Both of these methods have become least preferred choice after the development of more efficient and easier method like CRISPR/Cas9-based genome editing. The CRISPR/Cas9 strategy is considered a ground-breaking genome editing tool which has been successfully applied in many model and crop plants including tomato (Table 3). This technique involves RNA-guided engineered nucleases because of its simplicity, efficiency and versatility. In tomato, the CRISPR/Cas9 approach has been used to make modifications in the *ARGONAUTE7* (*SlAGO7*) gene. The mutation created in the *SlAGO7* was found to be stable in the subsequent generations of genome-edited tomato lines. The *SlAGO7* gene is responsible for the post-transcription degradation of *AUXIN RESPONSE FACTOR* (*ARF*) genes. Loss-of-function of this gene causes the conversion of compound leaves into needle-like or wiry leaves, hence silencing of this gene resulted in reduced levels of ARF and altered leaf phenotype in first and second generations successfully [7]. Furthermore, anthocyanins are a class of secondary metabolites which play a critical role in providing protection against biotic and abiotic stresses in plants [39,40]. 

Most of the genes involved in anthocyanin synthesis in plants have been identified. In addition to these genes, three families of transcription factors (TF) have been identified to play a role in the anthocyanin synthesis [40]. Under both ultraviolet (UV) and visible light, bZIP transcription factor, HY5 has been reported to have major effect in accumulation of anthocyanin. In order to identify TF in HY5 dependent and independent manner, a tomato cultivar ‘Indigo Rose’ bearing high anthocyanin containing purple tomatoes was selected and CRISPR/Cas9 was used to generate the mutants. Interestingly, transcriptome analysis of various tissues of *hy5* mutant lines revealed that eight TFs were found to be controlling anthocyanin biosynthesis independently [40]. Similarly, the CRISPR/Cas9 technique has been explored to develop high-quality and stress-resistant tomato lines [7,41,42,43,44].

## 3. Mutation Mapping Approaches

### 3.1. MutMap Approach

**Figure 1:**
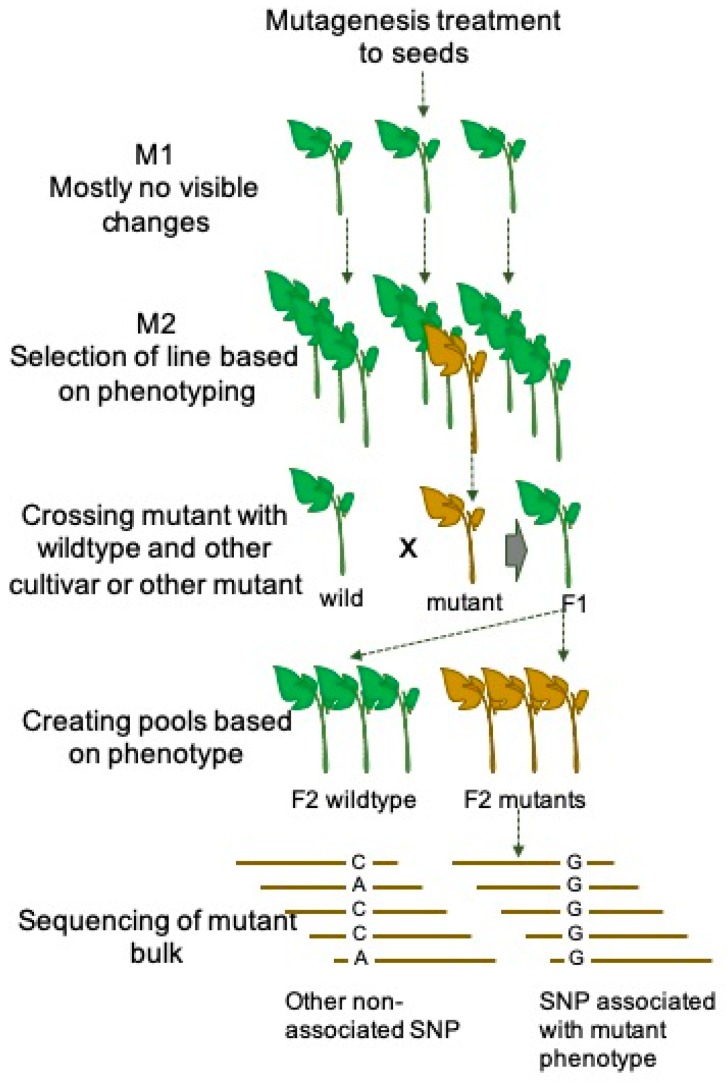
Schematic representation of atypical mutmap strategy exploring next generation sequencing to identify the causal mutations resulted into altered phenotype in plants. The mutmap strategy wasfirst demonstrated by Abe et al. (2012).

MutMap is a recently developed efficient and affordable forward genetic approach which is based on the high-throughput next-generation sequencing technique. MutMap has been demonstrated in rice for the first time to map mutations causing color change of leaves and semi-dwarf plant structure [59]. The major steps involved in the MutMap approach is illustrated in Figure 1. In a study conducted by [59], a rice cultivar was chemically mutagenized to develop a mutant population. The mutant lines mostly carry recessive mutations and such mutations do not impact phenotypic alteration. Therefore, the mutant lines need to be self-pollinated to obtain homozygous mutant populations in subsequent generations. Then the mutants with recessive mutation need to be identified based on the phenotypic screening and crossed with the wild type of the same cultivar. The obtained F1 hybrid plants need to self-crossed to obtain F2 generation. Furthermore, the bulk DNA of F2 population can be sequenced and mapped with reference genome of wild-type cultivar to identify the SNPs and insertions-deletions putatively related to the phenotype [59]. Due to the high applicability and reduced sequencing cost, MutMap can be utilized to identify desired genes with agronomic importance in lesser time [59]. The MutMap technique has recently been used in tomato to map mutations induced in the Micro-Tom cultivars [60]. A segregating F2 population have been developed by crossing the homozygous mutant line with a wild Micro-Tom, and separate pools of plants showing mutant and wildtype phenotypes have been sequenced with the NGS method to map the causal mutation. This study has demonstrated that the mapping of causal mutation can be performed within ten to twelve months in tomato with affordable cost.

### 3.2. MutChromSeq Approach

**Figure 2:**
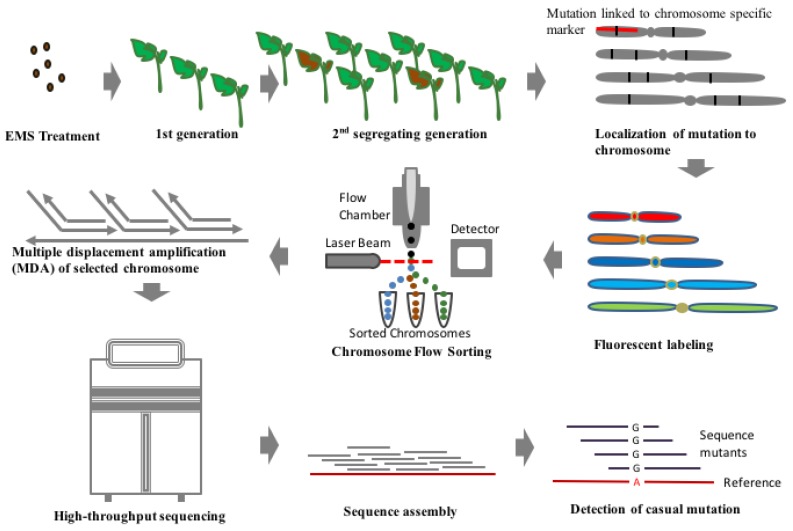
Generalised flowchart showing steps involved in the MutChromSeq approach which can be conveniently used for the mapping of casual mutation in plant species with larger genome size. To select chromosome for the sequencing, first mapping of confirmed mutant need to be done with markers known to be linked with each chromosome. Subsequently, chromosomes labelled with fluorescence dye can be sorted with flowcytometric techniques and sequenced selectively with high throughput techniques. Later analysis of sequencing data from the mutant line can be used to locate the casual mutation precisely similarly as described in Figure 1.

Finding a randomly induced mutation in a whole genome is like finding a needle in a haystack. However, with the advent of DNA sequencing, identifying genome-wide sequence variations has become easier. On the contrary, identification of sequence variation with high confidence using genome sequencing approach is easier only for plants with small genomes like Arabidopsis and rice while it is an expensive and computationally challenging approach for plants with larger genomes like barley and wheat. A recently discovered technique for rapid detection of casual mutation called MutChromSeq (Mutant Chromosome Sequencing) has been applied to identify novel genes in plants [61]. In this technique, classical mutagenesis is combined with chromosome flow sorting to filter out the desired region where we can find the desired gene. In this approach, the chromosomes are filtered out where particular gene is located on the basis of fluorescent marker tags (Figure 2). This is followed by sequencing of chromosomes of both mutant and non-mutants to find the desired gene of interest. This technique of isolating gene and DNA sequences can be applied to plants with complex and polyploidy genomes as well as genomes with large chromosome which are devoid of recombination and hence, reducing the need for recombination-based genetic mapping. This technique has been successfully employed in the case of barley and wheat for cloning of *Eceriferum-q* and *Pm2* gene [61]. This technique is applied to plants which are responsive for mutagenesis, the desired mutation results in significantly visible phenotypic change and prior information of the chromosome where the gene is located is available. Hence, it can be an inexpensive and robust technique of cloning of known unmanageable genes [61]. There is not a single report on MutChromSeq analysis in tomato, but its diploid nature and the availability of whole genome sequence make it easier to implement this technique for isolating and cloning desired genes in future.

### 3.3. Whole-Genome Sequencing (WGS)-Based Mapping

Once a high-quality reference genome available for any species, re-sequencing of many genotypes for the species become much easier, quicker and cheap. With the availability of whole genome sequence of *S. lycopersicum,* various attempts have been made for its re-sequencing using NGS to identify sequence variation among different cultivars [62]. Shirasawa et al. (2013) conducted re-sequencing of Micro-Tom mutants generated through EMS and gamma-ray radiation mutagenesis to locate the induced SNP and In Del mutations. To identify these mutations, paired-end reads by Illumina sequencing have been obtained for 8 Micro-Tom lines and mapped to the tomato reference genome. The study has successfully identified about a million SNPs and InDels [62]. Such whole genome re-sequencing approach generates a valuable resource of mutations to perform reverse genetic studies. The catalogue of identified mutation can be screened to sort out candidate variation possibly impacting gene function. For instance, SNPs causing non-synonymous changes can be detected using the genome annotation information. Considering the continuously reducing sequencing cost, projects aiming for the sequencing of the entire mutant population are anticipated in several crop species including tomato (Table 4). 

## 4. Tomato Mutant Resources

### 4.1. Genes that Make Tomatoes (http://zamir.sgn.cornell.edu/mutants/)

A resource of well characterized homozygous tomato mutants with the genetic background of cultivar M82 has been developed using EMS and fast-neutron mutagenesis approaches. To identify mutants for the agronomically important trait, extensive screening of around 13,000 M2 populations have been performed. Based on the phenotypic characterization, the mutant population was catalogued into 15 categories and 48 sub-categories. About 3417 mutations have been confirmed and characterized for the phenotype. Most of the mutations in the database are pleiotropic. The phenotypic data and images of the mutants are also made available on the database website. The mutant seeds can be obtained after requesting email with desired list of mutant codes.

### 4.2. LycoTILL (http://www.agrobios.it/tilling/)

LycoTILL is a database for tomato mutant lines generated through the EMS mutagenesis in genetic background of cv. Red Setter. The phenotypic data and images of confirmed mutants are available on-line in this database. The tomato cv. Red Setter was used because it is a highly productive processing variety and it has a vegetative cycle of about 110 days. The M2 mutant population has been phenotypically screened at different developmental stages and the recorded data then catalogued into 17 classes and 52 sub-classes. The database can be searched by phenotype, plant code and family name. About 6677 M2 and 5872 M3 families are comprised in this mutant collection and seeds of both the populations are made available.

## 5. The Red Setter TILLING Platform

The processing tomato cv. Red Setter was treated with EMS (0.7% and 1%) to apply TILLING to tomato. The DNA from 5221 M3 families was prepared to set TILLING platform and the M3 families were selected on the basis of seed abundance. About 66 induced point mutations were identified. Among these, SNPs were identified in both coding and non-coding genome. Among the coding region, both sense and missense mutations were found and missense mutations were predicated by the SIFT programme (Sorting Intolerant From Tolerant) to be deleterious for the activity of protein. This genetic resource could be used to discover high-throughput mutations (Minoia 2010).

### TOMATOMA (http://tomatoma.nbrp.jp/indexAction.do)

*TOMATOMA* is a database for tomato mutant lines in genetic background of Micro-Tom cultivar. The Micro-Tom is worldwide used as a model tomato cultivar in molecular biology research due to characteristic features like small plant size (10–20 cm height), short life cycle (fruit maturity in 70–90 days after sowing), ideal for indoor cultivation, can perform both interspecific and intraspecific cross proliferation, ease of proliferation, and highly efficient genetic transformation [78,79,80]. Therefore, to accelerate functional genomics studies, the mutant population of Micro-Tom has great importance. The mutant population have been developed using EMS and gamma radiations [80]. About 90,000 plants from M2 families were catalogued into 15 categories and 48 subcategories after confirming the mutant phenotypes and provided in a freely accessible database, TOMATOMA [80]. The aim of creating the TOMATOMA database was to provide the mutant seeds of Micro-Tom to the scientific community. These are available for a small fee on completion of a MTA (material transfer agreement). The images contained in the database are divided according to the phenotype and growth stage. The phenotype list includes images of seeds, plant size and habit, leaf morphology and color, flowering timing, inflorescence structure, flower morphology, color and size, fruit morphology, color and ripening, sterility and disease and stress response. The growth stage list includes images of germination, leaf production, side shoot, inflorescence, flowering fruit and ripening. The mutant resource provides, EMS mutagenesis lines, gamma irradiation-induced mutant lines, EMS and gamma irradiation-induced mutant lines, T-DNA tag lines and wild-type cultivars. All the three mutant resources described here need to be explored efficiently to accelerate the tomato improvement program (Table 5).

## 6. Limitations and Challenges for Mutagenesis in Tomato

Tomato has distinct characteristics such as the phylogenetic distance from other model plants as well not having any significant similarity in sequence information with other plant species. Therefore, even the availability of many efficient tools for both forward and reverse genetics is restricted in tomato. Even though around 30,000–40,000 genes have been identified and hundreds of tomato mutants are available, only a few links have been found for DNA sequences and mutants so far. Furthermore, high-throughput mutagenesis in tomato is challenging due to its short shelf life, long life cycle, time-consuming transformation and large-scale processing for seeds’ extraction immediately after the harvest from its jelly. Another big limitation is with transformation methods such as dipping and infiltration, which have been reported to be inefficient in tomato plants. With regards to efficient insertional mutagenesis in tomato, there are still no inhabitant transposons characterized in tomato. The development of gene editing and mapping approaches have enabled the progressions in the improvement of tomato, however, the above-mentioned limitations still require the implication of a combination of approaches and advancements towards tomato mutagenesis research.

## Figures and Tables

**Table 1:** List of significant studies describing induced mutagenesis efforts performed in tomato using different chemical and physical mutagens.

Tomato Cultivar	Mutagen	Concentration/Dose	Number of Mutants	Reason	Reference
Moneymaker	EMS (ethyl methane sulfonate)	60 mM	NA	Isolated *Adh1* null mutant of tomato	[9]
M82	EMS	0.5%	2552	For functional genomic studies	[10]
M82	Fast neutron	15 Gy	865	For functional genomic studies	[10]
*Lycopersicon esculentum* Mill.	Sodium azide	4 mM	31.07%	To improve the variety	[11]
*Lycopersicon esculentum* Mill.	EMS	1.5%	16	For resistance to *Orobanche ramosa* L.	[12]
Micro-Tom	Gamma-ray irradiation	300 Gy	6347	For functional genomics studies	[13]
Red Setter	EMS	1%	4500	To develop Red Setter TILLING platform	[14]
Red Setter	EMS	0.7%	8500	To develop Red Setter TILLING platform	[14]
M82	EMS		4759	For resistance to Potyvirus	[15]
Micro-Tom	EMS	1%	NA	For forward and reverse genetic studies	[1]

**Table 2:** Significant studies demonstrating use of insertional mutagenesis approach to induce mutations in tomato.

Tomato Variety/Cultivar	Insertional Mutagen	Target Gene/s	Transformation Method; Vector	Reference
cv. VF36	Dissociation transposable element with Ac3	NA	*Agrobacterium*-mediated; pBH2	[26]
cv. VFNT Cherry (LA 1221)	Maize transposable element *Ds1*	*polygalacturonase* (PG) and *dihydroflavonol 4-reductase* (DHFR) genes	*Agrobacterium*-mediated; pMON200	[24]
VFNT Cherry and New Yorker	Chimeric Ds element	Lc	*Agrobacterium*-mediated; pAL69 and pAL144	[27]
VF36 (LA490) and VFNT Cherry (LA1221)	Ac transposase and a chimeric Ds element	PG	*Agrobacterium*-mediated; pVCY1601 (VFNT Cherry)	[25]
cv. Micro-Tom	Activation-tagging technology	*ant1*	*Agrobacterium*-mediated; pSKI015	[28]
LA0315 and LA3899	*Rider* transposon	*SlMBP21*	*Agrobacterium*-mediated; pGEM-T Easy vector	[29]

**Table 3:** Details of genome-editing efforts performed to make site specific mutations in important genes in tomato.

Tomato Variety/Cultivar	Target Gene	Cas9 Promoter	Transformation Method; Vector	Effect	Reference
cv. M82	*ARGONAUTE7 (SlAGO7)*	U6 promoter	*Agrobacterium* *tumefaciens*-mediated; pAGM4723	First leaves of mutant plants having leaflets without petioles and later-formed leaves lacking laminae	[7]
*Solanum lycopersicum* var. M82	*SHORT-ROOT (SHR)* and *SCARECROW (SCR)*	35S promoter	*A. rhizogenes* transformation; pK7m24GW vector	Demonstrates that *SHORT-ROOT* and *SCARECROW* gene function is conserved between Arabidopsis and tomato	[45]
cv. M82	*SlCLV3*	*35S* promoter	*Agrobacterium*-mediated; pSC-A-amp/kan vector	Increased fruit size	[46]
cv. Micro-Tom	*ANT1*	35S promoter	*A. tumefaciens*-mediated; pCAMBIA1300	Resulted in intensely purple plant tissue	[38]
cv. Ailsa Craig	Ripening regulator, *RIN*	PcUbi4-2	*Agrobacterium* *tumefaciens*-mediated; pUC19_AtU6 oligo	Incomplete-ripening fruits were produced and confirming the important role of *RIN* in ripening	[47]
cv. Micro-Tom	*SlPDS* and *SlPIF4*	CaMV 35S and *AtUBQ* promoter	*Agrobacterium* *tumefaciens*-mediated; AtU6-sgRNA-AtUBQ-Cas9 and AtU6-sgRNA-2 × CaMV 35S-Cas9	Clear albino phenotypes for *psd* mutants	[48]
cv. M82	*SlBOP*	35S promoter	*Agrobacterium tumefaciens*-mediated; pSC-A-amp/kan vector	Causes pleiotropic defects, most notably simplification of inflorescences into single flowers, resembling tmf mutants	[49]
cv. Micro-Tom	*DELLA* and *ETR1*	*AtU6* promoter	*Agrobacterium* *tumefaciens*-mediated; pZK_FFCas9 and pUC19_AtU6oligo	Generated marker-free plants with homozygous heritable DNA substitutions	[43]
*S. lycopersicum* line FL8000	*SlDMR6-1*	2 × 35S promoter	*A. tumefaciens*-mediated; pPZP200	Confers broad-spectrum disease resistance	[50,51]
cv. M82 (LA3475) and *single flower truss* (*sft-7187*)	*SELF-PRUNING 5G* (*SP5G*)	35S promoter	*Agrobacterium tumefaciens*–mediated; Level 1 vector, pICH47751 and pICH47761and level 2 vector pAGM4723	Rapid flowering and enhance the compact determinate growth habit of field tomatoes	[52]
GCR758, a derivative of tomato cultivar Moneymaker	slmlo1	U6 promoter	*A. tumefaciens*-mediated; pAGM4723	Developed transgene-free powdery mildew resistant tomato variety, ‘Tomelo’	[53]
cv. Ailsa Craig	*SlMAPK3*	Ubi-H promoter	*Agrobacterium* -mediated cotyledon transformation; pYLCRISPR/Cas9 vector	Suggests that *SlMAPK3* is involved in drought response in tomato plants	[54]
cv. M82	*SlAGL6*		*Agrobacterium tumefaciens*-mediated; pRCS binary vector	Mutant is capable of fruit production under heat stress conditions	[55]
cv. Micro-Tom	*SlGAD2* and *SlGAD3*	AtU6 promoter	*Agrobacterium tumefaciens*-mediated; pZD_ AtU6_Hpger_Cas9_NPTII and pDeCas9_Kan	Increased GABA accumulation by 7 to 15 fold	[56]
cv. M82	*ALC* gene	*35S* promoter	*Agrobacterium tumefaciens*-mediated; pCAM1301	Recessive homozygous breeding elites with the character of long-shelf life were generated	[57]
cv. Micro-Tom and Ailsa Craig	*SlIAA9*	*2* × *CaMV35S* promoter	*Agrobacterium tumefaciens*-mediated; pEgP526-2A-GFBSD2	Morphological changes in leaf shape and seedless fruit	[51]
cv. Ailsa Craig (AC) and cv. Micro-Tom (MT)	Phytoene desaturase	Ubi promoter	*Agrobacterium*-mediated; pYLCRISPR/Cas9-slyPDS and -GABA vector	GABA accumulation enhance in both leaves and fruits	[58]

**Table 4:** List of significant efforts made towards mapping of causal mutation in tomato.

Tomato Cultivar	Mutant Trait	Mapping Method	Mutation (Position)	Reference
Moneymaker	gib-2	Linkage mapping	Chr 1	[63]
	gib-1		Chr 6	
	gib-3		Chr 7	
VF 11 and K93	*Rg-1*	Classical and RFLP (restriction fragment length polymorphism) mapping	Chr 3 (51 cM)	[64]
phytochrome A (phy A)-deficient fri mutants	fri	Classical map	Chr 10 (29 cM)	[65]
phyB1-deficient tri mutants	tri, hp-2	Classical and RFLP mapping	Chr 1 (33 cM)	[65]
Ailsa Craig and Liberto	Cnr (colorless nonripening)	RFLP and Linkage Analysis	Chr 2 (4.1-9.2 cM)	[66]
LA3179 and LA348	*Beta* (*B*) and *old-gold* (*og*)	Map-based cloning	Chr 6	[67]
LA2453, LA2455, and LA483	Green-ripe (Gr) and Never-ripe 2 (Nr-2)	Positional cloning	Chr 1 (2 cM)	[68]
Liberto and Ailsa Craig	Colorless non-ripening (*Cnr*)	Positional cloning	Chr 2 (13 kb)	[69]
LA3534, LA4074 and LA4076	*green-flesh* (*gf*)	Positional cloning	Chr 8 (45 cM)	[70]
Castlemart	**Od-2**	Genetic mapping	Chr 11 (16 cM)	[71]
*Solanum lycopersicum*	***lutescent 2 (l2)***	Linkage mapping	Chr 10L (7.5 cM)	[72]
*S. lycopersicum* (pe/pe) (LA2467) and *S. pimpinellifolium* (PE/PE) (LA1589)	***pe***	Genetic mapping	Chr 1 (424 kb)	[73]
P15C12 Micro-Tom glossy mutant × dwarf mutant from the M82 cultivar	***P15C12***	Genetic mapping	Chr 11 (4.84 Mb)	[74]
Micro-Tom	***pyp1***	Candidate gene approach with map-based cloning	Chr 1 (3.4 cM)	[75]
af (LA1049) × IL5-2 (LA4055)	***Af***	Map-Based Cloning	Chr 5	[76]
M82	***Nxd1***	Map-based cloning	Chr 12 (40-42 Mbp)	[77]

**Table 5:** Details of three major online resources, *Genes that make tomatoes*, *LycoTILL*, and *TOMATOMA* providing characterised mutant lines in tomato.

Information Resource	Genes that Make Tomatoes	LycoTILL	TOMATOMA
Genetic background	Inbred variety M82	cv. Red Setter	Micro-Tom
Mutagens	EMS and fast-neutrons	EMS	EMA and gamma-rays
Mutagen dosage	0.5% EMS and 12 Gy, 15 Gy Fast-neutron irradiation	0.7% and 1% EMS	0.3, 0.5, 1 and 1.5% EMS
Total M2/M3 families included	6000 EMS and 7000 fast neutron M2 families	6677 M2 and 5872 M3 families	4371 EMS and 6422 gamma-ray irradiated families
Total mutants catalogued	3417	-	1048
Total categories	15 primary and 48 secondary categories	17 classes and 52 sub-classes	15 major and 48 sub-categories
Managed by	Solanaceae resource	Metapontum Agrobios	National Bioresource Project Tomato (NBRP)
Seed request	Seeds obtained by sending email for the list of mutant codes	By signing of a Material Transfer Agreement (MTA) document	By sending 2 copies of MTA
